# Monthly Report of Diseases

**Published:** 1801-04

**Authors:** 


### MONTHLY REPORT of DISEASES;

Admitted under the Care of the PHYSICIANS of the FINSBURY DISPENSARY, St. John's Square, Clerkenwell.

From Feb. 20, to March 20, 1801.

| | No. of Cafes. | | No. of Cafes. |
|---|---|---|---|
| Chlorofis and Amenorrhœa | 25 | Infantile Difeafes - - - | 82 |
| Menorrhagia - - - - | 8 | Anafarca - - - - - - | 9 |
| Diarrhœa - - - - - - | 17 | Cephalæa - - - - - - | 2 |
| Cough and Dyfpnœa - - | 45 | Epilepfy - - - - - - | 6 |
| Phthifis Pulmonalis - - | 3 | Hyfteria - - - - - - | 3 |
| Cynanche Tonfillarum - | 5 | Afthenia - - - - - - | 48 |
| Eryfipelas - - - - - - | 16 | Hypochondriafis and Dyfpepfia - - - - - - | 39 |
| Continued Fever - - | 27 | | |
| Chronic Eruptions - - | 21 | | |

The cafes of amenorrhœa conftitute a confiderable proportion in the above lift. In a difeafe which is characterized by a general debility, and feldom attended by local inflammation, it is much to be wondered at, and to be lamented, that phyficians fhould have fuch frequent recourfe to the remedy of blood-letting ; which cannot fail, by weakening ftill farther, to aggravate all the morbid fymptoms it was intended to alleviate.

By no means is it uncommon for repeated venefections in fuch cafes to occafion an effufion upon the lungs, which foon terminates the fufferings, by terminating the exiftence of the patient.

That relaxation of body, that irritability and dejection of fpirits, and thofe various pains and uncomfortable feelings with which the young chlorotic female complains of being afflicted, are not to be relieved by bleeding, or any kind of lowering evacuants ; but, rather by the affiduous application of thofe mental and phyfical ftimuli, the direct tendency of which, is to induce a ftate of univerfal vigor and excitement.

Out of the great number of patients afflicted with this complaint, that have come under the care of the Reporter during the laft fifteen months of his practice at the Difpenfary, he recollects fcarcely a fingle inftance, in which fteel, in fome fhape or other, did not in a longer or fhorter time, accomplifh the object which he had in view.*

Cafes

* To fome it may feem remarkable, that fo many cafes of amenorrhœa fhould have fallen within the fphere of Difpenfary practice, as this is a difeafe that is, comparatively, feldom obferved amongft the lower claffes of fociety ; it is therefore neceffary to ftate, that although a confiderable number of our patients are from amongft almoft the pooreft of the community, there is ftill a large proportion of them, who, living as domeftics in opulent families, fhare in the luxuries of their fuperiors, and of courfe, in common with them, experience all the bad effects of good living. Befides, it is not fingular for governors of the charity, who are in a decent and even rather in

Cafes of fevers have confiderably decreafed in number during the laft month, whilft catarrhal and afthmatic affections have been much more extenfive in their prevalence, as well as troublefome in their fymptoms. In thefe complaints, efpecially when they attack the aged, little is to be done either by the apothecary's art, or by the fkill of the phyfician ; an accurate attention to *clothing* and *diet* is almoft all that can with advantage be recommended, unlefs in-indeed a change of air, which, however, amongft the lower claffes, is feldom practicable. The writer of this article has more than once feen a perfon, at an advanced period of life, afflicted with thefe catarrhal and afthmatic affections, fuddenly deprived, by the mercilefs hand of an empirical practitioner, of almoft all the few drops of blood that ftill lingered in his withered and nearly exhaufted veins. The diftended and over-charged veffels of the vigorous and the young may admit, and fometimes even require, a partial evacuation of their contents. But to take from an emaciated old man, bent under the weight of years, any part of that vital fluid, with which he is fo fcantily provided, is an act that would never be rafhly committed by any difcerning or intelligent practitioner. Fewer murders, perhaps, have been perpetrated by the fword than by the lancet. Next to the vaft fcythe of Time, fcarcely is there any weapon that has committed more cruel ravages than thofe which have been effected by this powerful, although minute inftrument of deftruction.

The inftances of hypochondriafis, recorded in our lift of difeafes, have in general occurred at an advanced period of life. There are few perfons indeed at an advanced period of life, in whom we may not detect, in a greater or lefs degree, the fymptoms of this difeafe. Objects in general, having loft in a good meafure their power of interefting, being no longer entertained by the amufements, nor engaged earneftly in the ferious occupations of life, and moft of thofe focial connections being broken which tended fomerly to divert his attention from himfelf, it is no wonder that the mind of an old man fhould often become occupied almoft entirely by the daily increafing infirmities of his body.

This will be ftill more likely to occur in thofe cafes where a perfon has been fo unfortunate as, in the earlier periods of his life, to expend in licentious and enfeebling pleafures, the whole of that corporeal vigor, part of which ought carefully to have been referved for the comfort and the fupport of age. A remark which might be illuftrated by the inftance of a celebrated perfonage in facred ftory, who, after having exhaufted the powers of his conan affluent condition, to convert the privilege which their annual fubfcription affords them, to the relief of their own wives and children, as well as of other friends, who ftand on the fame level of life with themfelves ! A circumftance which, although in various refpects highly conducive to the advantage of a young practitioner, cannot but appear glaringly inconfiftent with the nature and object of a charitable inftitution.

ftitution, by that unlimited debauchery to which his youth and manhood were devoted, at length complains, in the true temper of an hypochondriac, that—"*All is vanity and vexation of spirit.*"

*Red Lion Square.* J. R.

